# Enabling Episode-Level Transparency in Value-Based Care Through Large Language Model-Driven Provider Directories

**DOI:** 10.7759/cureus.102219

**Published:** 2026-01-24

**Authors:** Amol Kodan

**Affiliations:** 1 Public Health, Monroe University, New York City, USA

**Keywords:** artificial intelligence, episode-based care, healthcare transparency, large language models, provider directories, value-based care

## Abstract

Conventional provider directories, as a cornerstone interface, remain a critical yet structurally fragile component of the United States healthcare system, limiting transparency and constraining the effectiveness of value-based care (VBC). Conventional directory interfaces lack episode-level cost and risk context, rely on rigid search paradigms, and can contain inaccurate or incomplete information. These deficiencies hinder informed provider selection and weaken the operational impact of episode-based payment models. This study evaluates a large language model (LLM)-driven provider directory chatbot designed to support episode-based care navigation using strictly structured, synthetic cost and performance datasets. Four widely used LLMs, i.e., GPT-3.5-turbo, GPT-4o-mini, GPT-4o, and GPT-5.1, were assessed under identical deterministic conditions. Using 87 natural language test scenarios, we examined structured output validity, episode and risk-band identification, provider ranking accuracy, numeric fidelity, hallucination risk, abstention behavior, and operational performance. We further introduce a revised formulation of ranking correctness that explicitly treats accurate episode identification as a prerequisite for meaningful transparency. All models demonstrated consistently high episode identification accuracy, approaching 91%, though substantial variability was observed in downstream provider ranking reliability and numeric precision. Collectively, these preliminary findings suggest that LLM-enabled provider directories can meaningfully enhance transparency and the user experience within VBC settings, while highlighting specific performance dimensions that require optimization before large-scale deployment.

## Introduction

Episode-based payment models rely on accurate alignment between patient intent, clinically defined episodes of care, and provider-level cost and performance data. In operational settings, however, this alignment is rarely made explicit within provider directory interfaces. Instead, patients are required to infer episode relevance and value from fragmented provider attributes such as specialty labels or facility affiliation. This opacity represents a structural barrier to transparency and limits patients’ ability to act meaningfully on value-based care (VBC) incentives embedded in episode-based payment models [1,2]. In this study, episode-based care refers to grouping and paying for all services related to a specific condition or treatment over a defined period as a single unit (for example, all care associated with a knee replacement, including diagnostics/surgery/post-surgery care, all as one episode), while risk bands group patients by expected clinical complexity to help interpret costs fairly.

Recent advances in large language models (LLMs) offer the potential to address these limitations by enabling conversational interfaces that can interpret free-text queries and reason over structured clinical and financial data. Applied to provider directories, LLMs may facilitate direct mapping of patient intent to clinically appropriate episodes of care and support provider selection using episode-level cost and performance metrics [3]. However, healthcare applications impose substantially higher requirements than general conversational systems. Errors such as inaccurate episode attribution, hallucinated providers, or distorted cost outputs have the potential for inappropriate care decisions and real financial harm. As a result, evaluation frameworks must prioritize correctness, numeric fidelity, and safety rather than conversational fluency alone [3,4].

This study tests whether LLMs can meet clinical and financial safety expectations when using structured episode and provider performance data alone. To evaluate foundational capabilities safely, we use structured, synthetic datasets that allow controlled testing of episode identification, provider ranking, and numeric accuracy. This approach provides an important early step toward real-world deployment by demonstrating feasibility.

## Technical report

Models, experimental design, and output constraints

Four LLMs were evaluated under identical experimental conditions: GPT-3.5-turbo, GPT-4o-mini, GPT-4o, and GPT-5.1. All models were accessed through a unified API and operated using deterministic inference settings with zero temperature to ensure reproducibility and isolate structural model behavior. Each model independently processed the same set of 87 natural language test cases, and no conversational memory was retained between queries to isolate per-query behavior [3,4].

The chatbot operated under a fixed system prompt that explicitly prohibited the invention of providers, costs, or performance metrics and required exclusive reliance on provided CSV tables. Requests for in-network care were restricted to Tier 1 providers, and providers without matching episode-level performance data were excluded from ranked outputs. All responses were required to conform to a strict JSON schema followed by a plain language explanation, enabling automated validation, scoring, and safety checks consistent with healthcare deployment standards [4,5].

Across all models, structured output reliability was high. JSON parse success rates ranged from 96.6% to 100.0%, demonstrating that schema-constrained outputs are feasible even for complex, data-intensive prompts. Residual failures observed for GPT-4o and GPT-5.1 indicate that robust extraction, validation, and fallback logic remain necessary for safety-critical deployments, even under deterministic inference conditions [5-8].

Episode accuracy as the foundation of transparency

Episode identification accuracy was consistently high across all evaluated models, ranging from 89.7% for GPT-3.5-turbo and GPT-4o to 90.8% for GPT-4o-mini and GPT-5.1. This result is both technically and substantively significant. Correct episode mapping is the foundational step in episode-based navigation because it determines which cost and performance data are surfaced to members and which providers are considered relevant within VBC frameworks [1,2].

Risk band identification exhibited substantially greater variation, with accuracy ranging from 39.1% to 74.7% across models. This divergence indicates that improvements in general language modeling do not automatically translate into reliable extraction of structured risk attributes. From a systems perspective, this finding underscores the need for explicit risk handling logic or clarification pathways in episode-based directories, rather than relying on generative inference alone [5,6].

Traditional ranking metrics such as Top-1 correctness and Normalized Discounted Cumulative Gain (NDCG) implicitly assume correct episode identification. In member-facing episode navigation, this assumption does not hold. A ranking that is modestly suboptimal but grounded in the correct episode can still deliver meaningful transparency, whereas a perfectly ranked list for the wrong episode is inherently misleading. Accordingly, this study reframes ranking evaluation to explicitly weight episode correctness as a gating condition, aligning evaluation with real member experience and protection goals [2,4].

From a member perspective, this reframing reflects a conceptual shift in directory interaction. Traditional directories require manual translation of care needs into filters and provide no episode-level cost context. An LLM-driven directory that reliably identifies episodes and constrains outputs to authoritative data can surface cost-ranked, in-network provider options with minimal effort. This reduces cognitive burden, improves trust, and strengthens alignment between member behavior and VBC incentives [1,2,8].

Results emphasizing episode-weighted transparency

The evaluation included 87 natural-language test cases per model. Summary metrics were computed as rates with 95% confidence intervals for proportions and bootstrap confidence intervals for means. Results are reported in aggregate and with language stratification for English (n = 71) and non-English (n = 16) subsets.

All evaluated models demonstrated high structured output validity. JSON parse success ranged from 96.6% to 100.0%, confirming that strict schemas and machine-readable outputs are feasible for provider directory chatbots operating over large tabular inputs. Residual failures observed for GPT-4o and GPT-5.1 indicate that production deployments require robust schema validation and safe fallback behaviors [5,8].

Episode identification accuracy was consistently high across all models, establishing that contemporary LLMs can reliably map free-text member intent to episode constructs, a prerequisite for episode-based cost comparison in VBC settings [1,2].

Risk-band identification showed greater dispersion. While risk stratification influences cost interpretation, errors at this stage occur downstream of episode identification and can be mitigated by providing clarification prompts or by using deterministic logic. Episode accuracy, therefore, represents the dominant driver of whether a directory interaction yields meaningful transparency [5,6].

For clarity, episode accuracy reflects whether the model correctly identifies the clinically appropriate episode of care from a natural language query, which serves as a prerequisite for meaningful provider comparison. Top-ranked correctness captures whether the highest-ranked provider is the correct choice within the identified episode, while NDCG at rank three (NDCG@3) measures the overall quality of the top three ranked providers by weighting higher-ranked correct providers more heavily than lower-ranked ones. In the episode weighted formulation, episode correctness acts as a binary gating condition; α represents a tunable parameter that controls the relative emphasis placed on exact top rank correctness versus broader ranking quality, ensuring that no ranking credit is assigned when the episode itself is incorrect.

The Episode Weighted Ranking Accuracy metric is defined as:

$\text{Episode Weighted Ranking} = \mathbf{1}(\text{Episode Correct}) \times \left[ \alpha \cdot \mathbf{1}(\text{Top Ranked Provider Correct}) \cdot (1 - \alpha) \cdot \mathrm{NDCG@3} \right]$

Where episode correctness is a binary prerequisite and ($\alpha$) controls the relative emphasis on exact top-ranked correctness versus overall ranking quality. This formulation ensures that no ranking credit is assigned when the episode is incorrect, aligning the metric with transparency and member protection goals (Table 1).

**Table 1:** Model performance comparison across episode accuracy and ranking metrics. NDCG = Normalized Discounted Cumulative Gain

Model	Episode accuracy (%)	Top-ranked correct (%)	NDCG@3
GPT-4o	89.7	59.8	0.632
GPT-5.1	90.8	48.3	0.601
GPT-4o-mini	90.8	41.4	0.539
GPT-3.5-turbo	89.7	42.5	0.499

When episode correctness is treated as foundational, all models exhibit stronger transparency-aligned behavior than raw ranking metrics alone would suggest. Even models with moderate top-ranked accuracy consistently place members within the correct episode context, which substantially reduces the risk of irrelevant or misleading cost comparisons.

Numeric fidelity further differentiates model suitability for transparency use cases. Numeric exact match measures whether the returned episode-level cost and quality metrics exactly match the corresponding rows in the episode performance table. Hallucination rate captures unsupported provider identifiers or provider episode risk combinations, while abstention accuracy captures the model’s ability to correctly withhold responses when required data are unavailable or constraints are violated (Table 2).

**Table 2:** Numeric fidelity, hallucination, and safety metrics.

Model	Numeric exact match accuracy (%)	Hallucination rate (%)	Abstention accuracy (%)
GPT-4o	79.3	10.3	92.0
GPT-4o-mini	58.6	26.4	74.7
GPT-5.1	40.2	43.7	57.5
GPT-3.5-turbo	29.9	47.1	61.0

The strongest performing model combined high numeric fidelity with low hallucination and high abstention correctness. This profile is particularly important for member-facing transparency tools. Incorrect cost values or unsupported provider recommendations undermine trust and can expose members to unexpected financial risk. Models with elevated hallucination rates may still be useful for conversational intake or clarification, but they require stronger guardrails and post-generation verification before presenting cost and quality outputs [5,8].

Operational performance was also measured to assess feasibility for real-world deployment. Average latency ranged from approximately 4.6 seconds to 12.2 seconds per query, with total token usage clustered between 6,463 and 6,863 tokens across models. Token similarity reflects the inclusion of full CSV tables in the evaluation prompt. In production systems, the retrieval of only episode-relevant rows would be expected to materially reduce latency and inference cost [4].

Language stratification results suggest that multilingual robustness is achievable but not guaranteed. The strongest model maintained relatively high numeric fidelity in English and showed a modest decline in non-English queries, accompanied by a small increase in hallucination. These results underscore the need for language-specific evaluation before broad deployment [6,8].

Taken together, the results demonstrate that episode accuracy is already strong across contemporary LLMs and serves as a powerful anchor for transparency. When combined with appropriate ranking, numeric fidelity, and safety controls, LLM-driven provider directories can meaningfully improve member experience by reducing cognitive burden, clarifying cost trade-offs, and supporting value-aligned provider selection [1-5,8].

## Discussion

Discussion and implications for value-based care

This evaluation demonstrates that LLM-driven provider directory chatbots can materially improve transparency and member experience in episode-based VBC when episode accuracy is treated as the foundational requirement. Across all evaluated models, episode identification accuracy approached 91%, a level of performance that is both technically significant and operationally meaningful. Correct episode mapping ensures that members are placed within the appropriate clinical and financial frame of reference before any cost comparison or provider ranking occurs [1,2,9].

By reframing ranking correctness to explicitly weight episode accuracy as a gating condition, this study aligns evaluation metrics with real member experience. A ranking that is imperfect but grounded in the correct episode still supports informed decision-making, while any ranking produced for an incorrect episode undermines transparency entirely. This distinction is critical in healthcare contexts, where relevance and safety outweigh marginal gains in ranking precision [2,8,10].

The results also highlight important tradeoffs across models. While some models exhibited strong ranking behavior, elevated hallucination rates and lower numeric fidelity limited their suitability for member-facing transparency use cases. Conversely, the strongest performing model combined high episode accuracy, high numeric exact match, low hallucination, and strong abstention behavior. This profile illustrates that transparency is not a single metric, but an emergent property of correctness, constraint adherence, and disciplined failure behavior [5,8,11].

Member experience impact

From a member perspective, the transition from traditional provider directories to episode-aware conversational interfaces represents a structural improvement rather than an incremental usability enhancement. Traditional directories require members to translate care needs into filters, navigate long provider lists, and infer value without explicit cost context. This process imposes a high cognitive burden and frequently results in suboptimal provider selection [1,7].

In contrast, an LLM-driven directory that reliably identifies episodes and constrains outputs to authoritative data can accept natural language intent, automatically apply network and episode constraints, and surface a small set of cost-ranked providers with plain language explanations. This reduces effort, increases confidence, and enables members to engage directly with the cost and quality signals embedded in value-based payment models [2,9].

Importantly, the study shows that these benefits are already achievable with current generation models, provided that systems are designed around episode accuracy, numeric fidelity, and safety rather than conversational breadth [3,4].

Governance, safety, and deployment considerations

The findings reinforce that LLMs should not be treated as standalone decision makers in provider directories. Several safety-critical functions are better handled through deterministic system design. Network filtering, exclusion of providers without episode performance data, numeric validation, and abstention triggers should be enforced outside the model wherever possible. The LLM’s primary value lies in intent interpretation and explanation, not in unconstrained reasoning over cost data [4,5,10].

High abstention correctness emerged as a key differentiator among models. In VBC, declining to answer when data are insufficient is preferable to presenting unsupported recommendations. Systems that prioritize apparent helpfulness over correctness risk eroding trust and exposing members to unexpected financial outcomes [5,8,11].

Operational metrics suggest that latency and token usage are manageable for real-world deployment, particularly when combined with retrieval strategies that limit prompts to episode-relevant data. Schema-constrained outputs and validation pipelines further support auditability and regulatory compliance [4,7,11].

Opportunities for further improvement

While the results are encouraging, several opportunities exist to further enhance transparency and member experience. One high-impact extension is the integration of provider availability data, such as near-term appointment slots. Episode-based cost transparency alone may still leave members choosing between providers who are unavailable for weeks or months. By incorporating availability signals, directories can surface providers who are both high value and accessible within a relevant time horizon [7,9].

Additional improvements include explicit risk clarification flows, multi-objective ranking that incorporates quality metrics alongside cost, and expanded multilingual evaluation to ensure equitable performance across member populations. Continued advances in model training and fine-tuning, combined with stronger retrieval and validation architectures, are likely to further reduce hallucination and improve numeric fidelity [5,6,12].

Limitations

This evaluation has several limitations. First, this study uses synthetic data to enable controlled, reproducible evaluation without exposure to protected health information. While the dataset preserves realistic distributions and constraints, real-world claims data may introduce additional complexity. The evaluation also focuses on single-turn interactions. Multi-turn clarification flows may improve performance, but were outside the scope of this analysis. While appropriate for methodological evaluation, synthetic data may not capture the full heterogeneity, noise, and missingness present in real-world VBM environments. Second, models were evaluated under deterministic inference settings, which improve reproducibility but may underestimate response variability encountered in production systems. Third, risk band classification was assessed as an exact match task, although clinical risk stratification may warrant probabilistic or ordinal interpretations in operational use. Fourth, latency and token usage reflect prompts containing full tabular context; production implementations using targeted retrieval are likely to exhibit different performance profiles. Representative errors in this study included plausible but incorrect episode cost values and cases where a valid provider was incorrectly associated with an unsupported episode or risk band, indicating structural rather than conversational failure modes. Finally, the non-English subset was small, and conclusions regarding multilingual robustness should be interpreted as directional [4,6,9-12]. All conclusions related to deployment are conditional and forward-looking, and validation using real-world claims data is required before operational use.

The strongest performing model combined high numeric fidelity with low hallucination and high abstention correctness. This profile is particularly important for member-facing transparency tools. Incorrect cost values or unsupported provider recommendations undermine trust and can expose members to unexpected financial risk. Models with elevated hallucination rates may still be useful for conversational intake or clarification, but they require stronger guardrails and post-generation verification before presenting cost and quality outputs. This study did not evaluate downstream clinical outcomes or behavioral responses, which are necessary to determine whether improved transparency translates into sustained engagement or cost-effective care selection. Future work should validate these findings in live deployments with human-in-the-loop safeguards [2-4].

## Conclusions

This study provides preliminary evidence that LLM-driven provider directory chatbots can meaningfully advance transparency and improve member experience in episode-based VBC. By demonstrating consistently high episode identification accuracy across models and reframing ranking correctness to prioritize episode accuracy, the findings highlight a critical strength of contemporary LLMs that has been underemphasized in prior evaluations. When combined with strong numeric fidelity, low hallucination rates, appropriate abstention behavior, and deterministic guardrails, LLM-driven directories can transform provider search from a fragmented, opaque process into a guided, value-aligned decision support experience. With continued model advancement and integration of additional signals such as provider availability, these systems have the potential to become a core infrastructure component supporting transparent, member-centered, and value-based care.
